# Dynamics of the COVID-19 Clinical Findings and the Serologic Response

**DOI:** 10.3389/fmicb.2021.743048

**Published:** 2021-10-07

**Authors:** Ahmadreza Niavarani, Hossein Poustchi, Amaneh Shayanrad, Maryam Sharafkhah, Zahra Mohammadi, Fariborz Mansour-Ghanaei, Farahnaz Joukar, Gholamreza Roshandel, Ahmad Hormati, Reza Ghadimi, Khosro Sadeghniiat-haghighi, Alireza Abdollahi, Masoud Mardani, Ayad Bahadorimonfared, Shahla Ghanbari, Alireza Delavari, Abbas Vosoogh-Moghaddam, Mohammad Zamani, Farzin Roozafzai, Saba Alvand, Maryam Darvishian, Reza Malekzadeh

**Affiliations:** ^1^Digestive Oncology Research Center, Digestive Diseases Research Institute, Shariati Hospital, Tehran University of Medical Sciences, Tehran, Iran; ^2^Liver and Pancreatobiliary Diseases Research Center, Digestive Diseases Research Institute, Shariati Hospital, Tehran University of Medical Sciences, Tehran, Iran; ^3^Sasan Alborz Research Center, Masoud Clinic, Tehran, Iran; ^4^Division of Gastroenterology & Hepatology, Gastrointestinal and Liver Diseases Research Center, Guilan University of Medical Sciences, Rasht, Iran; ^5^Golestan Research Center of Gastroenterology and Hepatology, Golestan University of Medical Sciences, Gorgan, Iran; ^6^Gastrointestinal and Liver Diseases Research Center, Iran University of Medical Sciences, Tehran, Iran; ^7^Gastroenterology and Hepatology Disease Research Center, Qom University of Medical Sciences, Qom, Iran; ^8^Social Determinants of Health Research Center, Health Research Institute, Babol University of Medical Sciences, Babol, Iran; ^9^Occupational Sleep Research Center, Tehran University of Medical Sciences, Tehran, Iran; ^10^Department of Pathology, School of Medicine, Imam Hospital Complex, Tehran University of Medical Sciences, Tehran, Iran; ^11^Infectious Diseases and Tropical Medicine Research Center, Shahid Beheshti University of Medical Sciences, Tehran, Iran; ^12^Department of Health and Community Medicine, Faculty of Medicine, Shahid Beheshti University of Medical Sciences, Tehran, Iran; ^13^Department of Health Education and Health Promotion, Deputy for Health Affairs, Shahid Beheshti University of Medical Sciences, Tehran, Iran; ^14^Community Medicine Specialist, Governance and Health Research Group, Neuroscience Research Institute, Tehran University of Medical Sciences and Health Services, Tehran, Iran; ^15^Digestive Diseases Research Center, Digestive Diseases Research Institute, Tehran University of Medical Sciences, Tehran, Iran; ^16^Cancer Control Research, BC Cancer Research Centre, Vancouver, BC, Canada; ^17^Digestive Diseases Research Center, Digestive Diseases Research Institute, Shariati Hospital, Tehran University of Medical Sciences, Tehran, Iran

**Keywords:** anti-SARS-CoV-2 antibody, COVID-19, persistent disease, antibody sustain, ABO blood group antigen, respiratory symptoms, gastrointestinal symptoms

## Abstract

The factors affecting the dynamics of lengthening of symptoms and serologic responses are not well known. In order to see how the serologic responses change in relation to the clinical features, we selected a group of 472 adults with a positive IgM/IgG antibody test result from a baseline study of the anti-SARS-CoV-2 seropositivity, assessed their COVID-19 and past medical histories, and followed them up in about 3 months. Nearly one-fourth of the subjects were asymptomatic at the baseline; 12.8% subjects became symptomatic at the follow-up (FU) when 39.8% of the subjects had some persisting symptoms. At the baseline, 6.1% showed anti-SARS-CoV-2 IgM positive, 59.3% only for IgG, and 34.5% for both. At the FU, these figures declined to 0.6, 54.0, and 4.4%, respectively, with the mean IgM and IgG levels declining about 6.3 and 2.5 folds. Blood group A was consistently linked to both sustaining and flipping of the gastrointestinal (GI) and respiratory symptoms. The baseline IgM level was associated with GI symptoms and pre-existing cirrhosis in multivariate models. Both of the baseline and FU IgG levels were strongly associated with age, male, and lung involvement seen in chest computed tomography (CT)-scan. Finally, as compared with antibody decayers, IgM sustainers were found to be more anosmic [mean difference (MD): 11.5%; *P* = 0.047] with lower body mass index (BMI) (MD: 1.30 kg/m^2^; *P* = 0.002), while IgG sustainers were more commonly females (MD: 19.2%; *P* = 0.042) with shorter diarrhea duration in the FU (MD: 2.8 days; *P* = 0.027). Our findings indicate how the anti-SARS-CoV-2 serologic response and COVID-19 clinical presentations change in relation to each other and basic characteristics.

## Introduction

The coronavirus disease of 2019 (COVID-19) was first reported in an outbreak in late 2019 in Wuhan City, China, with the novel severe acute respiratory syndrome coronavirus-2 (SARS-CoV-2) as the underlying pathogen (Chan et al., 2020; Hung et al., 2020). It was rapidly spread all over the globe, making a Public Health Emergency of International Concern (PHEIC; Chan et al., 2020). According to the John Hopkins University of Medicine^1^, there have been more than 215 million confirmed cases of COVID-19 by August 30, 2021, leading to about 4.5 million deaths. With a deadly viral infection that rapidly spreads both clinically and subclinically, it would be very critical to characterize the modes of immune response and the duration of protection against COVID-19. Although the T-cell-mediated immune response appears to be very robustly present in individuals convalescing from disease (Le Bert et al., 2020) and even pre-existing T cell immunity induced by circulating alpha- and beta-human coronaviruses is important in younger adults (Saletti et al., 2020), it is much easier to assess the humoral immune response in COVID-19. In fact, the humoral responses to the spike (S) and nucleocapsid (N) SARS-CoV-2 proteins were reported as faithful indicators of 6-month protection against COVID-19 (Lumley et al., 2021). The fairly large 40-kDa N protein makes it a strong and good candidate for serologic diagnosis (Kumar et al., 2020). The length of the active disease as well as the immune response appears to be more complicated. In a study of hospitalized COVID-19 patients, nearly 15% were found to be persistently positive for reverse transcription- polymerase chain reaction (RT-PCR) test between days 41 and 60 of discharge (Cento et al., 2020), raising questions about the capacity of the immune system to completely eradicate SARS-CoV-2 in some individuals. Intriguingly, longitudinal analysis of antibody responses to SARS-CoV-2 proposed a rapid resolution of disease symptoms correlated with sustaining IgG production (Chen et al., 2020). However, this has not yet been corroborated by other studies, and the nature of the symptoms was not investigated in more detail. On the other hand, many patients do not show full recovery 12 weeks or more after COVID-19, defined as the post-COVID-19 syndrome (Sivan and Taylor, 2020). In order to see how the serologic responses alter in relation to the clinical features, we assessed the changes in clinical findings and the serologic response to SARS-CoV-2 for 3 months, and investigated whether they were correlated with each other as well as with basic characteristics and medical conditions.

## Materials and Methods

### Ethical Statement

Both the study proposal and protocol were approved by the Ethics Committee of the Tehran University of Medical Sciences (reference number: IR.TUMS.VCR.REC.1399.308).

### Patients

In this population-based longitudinal study, we selected all 472 subjects with a seropositive response, IgM and/or IgG, to the SARS-CoV-2 from a larger study of 8,902 individuals (Poustchi et al., 2021) in order to assess the dynamics of the serological response in relation to various factors, including the clinical presentation. In brief, all subjects were randomly selected from both the general population and the individuals with a high risk of occupational exposure to SARS-CoV-2 (e.g., health care workers, taxi drivers, and cashiers) from five large Iranian cities including Tehran, Qom, Gorgan, Babol, and Rasht, with no particular exclusion criteria (Poustchi et al., 2020). These subjects included 252 men aged between 19 and 77 years old, as well as 220 women aged between 23 and 87 years old. The baseline interview and blood sampling were conducted between July 14 and August 1, 2020 in the cities, including five centers or universities across Tehran. At the time, the B.4 and B.1^∗^ lineages of the SARS-CoV-2 were predominant in the country, increasingly accompanied by the D614G mutation (Fattahi et al., 2021). Participants’ demographic characteristics, medical histories, recent COVID-19-related symptoms, and COVID-19-related exposures were recorded in pertinent questionnaires. A sampling of the peripheral blood allowed later anti-SARS-CoV-2 IgM and IgG antibody testing of participants’ sera using PT-SARS-CoV-2.IgM-96 and PT-SARS-CoV-2.IgG-96 kits (Pishtaz Teb, Tehran, Iran). All enzyme-linked immunosorbent assays (ELISA) targeting the N-protein of SARS-CoV-2 were conducted as recommended by the manufacturer. Immunoglobulin levels were further classifed as “negative (<1.10), 1 + (1.10–2.99), 2 + (3.00–5.99), 3 + (6.00–11.99), and 4 + (≥12.00).” All 472 participants with either positive anti-SARS-CoV-2 IgM or positive anti-SARS-CoV-2 IgG antibodies were enrolled in a follow-up (FU) assessment after about 3 months, including a questionnaire regarding incident events, as well as 1 s blood sampling for anti-SARS-CoV-2 serologic testing.

Various self-reported symptoms were classified as follows in order to dissect the impact of disease on various organs: systemic symptoms including fever, chills, headache, weakness, myalgia, arthralgia, convulsions, and loss of consciousness; respiratory symptoms including dry cough, cough with sputum, sore throat, dyspnea, anosmia, dysgeusia, conjunctivitis, rhinorrhea, and chest pain; and gastrointestinal (GI) symptoms including diarrhea, nausea, vomiting, and GI bleeding. Both IgM and IgG declines were quantified over time by calculating the quotient of the FU immunoglobulin level divided by the baseline one, regarded as the “antibody durability index” (ADI). Based on the ADI, all individuals were classified into antibody “decayers” (ADI < 1) or “sustainers” (ADI ≥ 1). Those asymptomatic subjects at the baseline who developed symptoms at the FU were deemed as flipping to symptomatic, and those with at least 50% of the symptoms at the FU as compared with the baseline were considered to be persistently symptomatic, whether globally or in symptom classes (systemic, respiratory, and GI).

### Statistical Analysis

We used D’Agostino & Pearson normality test to assess the normal distribution of the data. Pearson regression analysis was used to assess the correlations between the immunoglobulins and basic characteristics, including age, sex, and ABO blood group, as well as testing conditions, including days since diagnosis, symptom classes, and pre-existing conditions. Multivariate linear regression analysis was performed to assess the overall impact of the above-mentioned factors on IgM and IgG levels, using those variables with a significance level of less 0.05 or less in univariate analysis. For those parameters without a normal distribution, unpaired two-tailed Student’s *t*-test with Welch’s correction was used to compare the mean level of the immunoglobulins among various subgroups of age, sex, ABO blood group, and chest computed tomography (CT) scan positivity. It was also used to compare the frequency of symptomatic cases in antibody sustainers and decayers, and various characteristics between those who flipped to symptomatic at FU and those who did not, and between those with sustaining symptoms at FU and those without. All analyses were performed using IBM SPSS software v.22 (IBM Corp., Armonk, NY, United States) with a significance level of 0.05.

### Data Access

The data is available through the request to co-responder author.

## Results

### Baseline Findings

**FIGURE 1:**
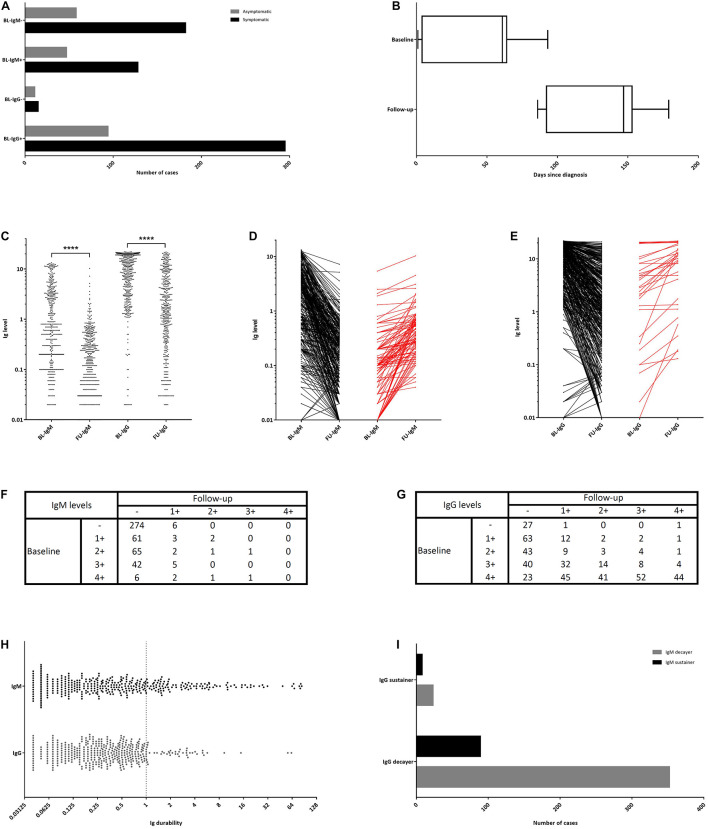
The time course of the baseline (BL) and follow-up (FU) blood sampling and the dynamics of the anti-SARS-CoV-2 seropositivity and symptom changes. **(A)** The number of symptomatic and asymptomatic subjects in each IgM/IgG negative/positive subgroup. **(B)** The time-course of the baseline as well as the FU interview and serologic testing. **(C)** IgM and IgG antibody levels at the baseline and FU. **(D)** The extent of antibody level change in IgM decayers (black) and IgM sustainers (red). **(E)** The extent of antibody level change in IgG decayers (black) and IgG sustainers (red). **(F)** The extent of IgM level change in semiquantitative form. **(G)** The extent of IgG level change in semiquantitative form. **(H)** IgM and IgG durability. **(I)** The number of IgM and IgG sustainers and decayers. (– [negative]: <1.10, 1+: 1.10–2.99, 2+: 3.00–5.99, 3+: 6.00–11.99, and 4+: ≥12.00). ****unpaired two-tailed Student’s *t*-test, *P* < 0.0001.

A total of 472 participants with positive anti-SARS-CoV-2 IgM and/or IgG antibodies were selected for this study. Subjects were 19 to 87 years old (median: 43 years), including 252 (53.4%) men with a median age of 45 years, and 220 (46.6%) women with a median age of 41 years (Table 1). The median time from clinical onset of COVID-19 to the baseline testing was 61 days (interquartile range [IQR]: 4–64; Figure 1B). Overall, 352 (74.6%) subjects experienced at least one symptom. This ratio was not discrepant between those with positive and negative IgM responses, 73.1 and 75.8%, respectively (Figure 1A). However, a trend for a higher ratio of symptomatic subjects was seen in those showing the positive response (75.8%) compared with the negative responders (57.7%, *P* = 0.059; Figure 1A). On the other hand, 120 (25.4%) subjects were reported to be asymptomatic. At the baseline, 29 (6.1%) subjects were seen to be positive only for anti-SARS-CoV-2 IgM antibody, 270 (57.2%) only for anti-SARS-CoV-2 IgG, and 163 (34.5%) for both the antibodies. Median baseline anti-SARS-CoV-2 IgM and IgG levels were 0.60 (IQR: 0.14–3.20) and 9.60 (IQR: 3.19–18.45), respectively (Figure 1C). At the baseline, 57 (12.1%) subjects had a history of hospitalization for COVID-19, of whom 40 (8.5%) had shown positive CT-scans and 20 (4.2%) shown positive PCR tests. Both the mean baseline anti-SARS-CoV-2 IgM (mean difference [MD]: 1.60, *P* = 0.013) and IgG levels (MD: 5.22, *P* < 0.001) were found to be higher in hospitalized subjects as compared with the non-hospitalized.

**TABLE 1:** Basic characteristics and anti-SARS-CoV-2 serologic profile of 472 COVID-19 patients.

**Characteristics**	**Categories**			Baseline*	Follow-up*
Sex	Female			220 (46.6)	
	Male			252 (53.4)	
Age group (years)	<20			8 (1.7)	
	20–29			42 (8.9)	
	30–39			131 (27.8)	
	40–49			167 (35.4)	
	50–59			91 (19.3)	
	60–69			24 (5.1)	
	>70			9 (1.9)	
Occupation	Health care workers			222 (47.0)	
	Non-health care workers			250 (53.0)	
Hospitalization	No			415 (87.9)	
	Yes	CT scan −	PCR −	7 (1.5)	
			PCR +	10 (2.1)	
		CT scan +	PCR −	30 (6.4)	
			PCR +	10 (2.1)	
Anti-SARS-CoV-2 antibody	IgM	− (<1.10)		280 (59.3)	448 (94.9)
		1 + (1.10–2.99)		66 (14.0)	18 (3.8)
		2 + (3.00–5.99)		69 (14.6)	4 (0.8)
		3 + (6.00–11.99)		47 (10.0)	2 (0.4)
		4 + (≥12.0)		10 (2.1)	0 (0.0)
	IgG	− (<1.10)		29 (6.1)	196 (41.5)
		1 + (1.10–2.99)		82 (17.4)	99 (21.0)
		2 + (3.00–5.99)		58 (12.3)	60 (12.7)
		3 + (6.00–11.99)		98 (20.8)	66 (14.0)
		4 + (≥12.0)		205 (43.4)	51 (10.8)

*Numbers represent “count (percentage).”

*−: negative, +: positive.*

*CT: computed tomography, IgG: immunoglobulin G, IgM: immunoglobulin M, PCR: reverse transcription polymerase chain reaction, and SARS-CoV-2: novel severe acute respiratory syndrome coronavirus-2.*

### Follow-Up Findings

Follow-up interviews and serologic testing were conducted for all subjects after a median of 87 days (IQR: 84–91) from the baseline (Figure 1B). The median time from clinical onset of disease to FU testing was 147 (range: 86–179) days. Three (0.6%) subjects were positive only for IgM, 255 (54.0%) only for IgG, and 21 (4.4%) for both anti-SARS-CoV-2 antibodies; and, 193 (40.9%) subjects found to be negative for both the immunoglobulins. Anti-SARS-CoV-2 IgM level decreased from the mean (±standard deviation [SD]) baseline level of 2.24 (±3.23) to 0.35 (±0.76) at the FU, showing a 6.3 fold decline (Figure 1C). Similarly, the anti-SARS-CoV-2 IgG level decreased from the baseline level of 10.46 (±7.35) to 4.12 (±5.29), exhibiting a 2.5 fold decline. Median FU anti-SARS-CoV-2 IgM and IgG levels were 0.16 (IQR: 0.03–0.41) and 1.61 (IQR: 0.41–5.97), respectively. Furthermore, median FU anti-SARS-CoV-2 IgM levels in subjects with and without the history of hospitalization were 0.25 (IQR: 0.10–0.55) and 0.17 (IQR: 0.05–0.45), respectively; and, the corresponding median IgG levels were 7.08 (IQR: 2.23–12.84) and 1.44 (IQR: 0.37–4.94), respectively. Mean FU anti-SARS-CoV-2 IgG levels were higher in the hospitalized subjects compared with the non-hospitalized ones (MD: 4.53, *P* < 0.001); but, the observed difference in the IgM levels was not statistically significant (MD: 0.21; *P* = 0.24). Ninety-seven (20.6%) participants showed increased anti-SARS-CoV-2 IgM levels (IgM sustainers; Figures 1D,H), while only 31 (6.6%) had increased IgG levels (IgG sustainers; Figures 1E,H). Figures 1F,G show details on how the baseline IgM and IgG levels changed at the FU. Thirty-one subjects showed IgG sustaining, of whom eight sustained IgM levels as well. On the other hand, 441 participants showed IgG decay, of whom 352 decayed their IgM levels as well (Figure 1I).

**FIGURE 2:**
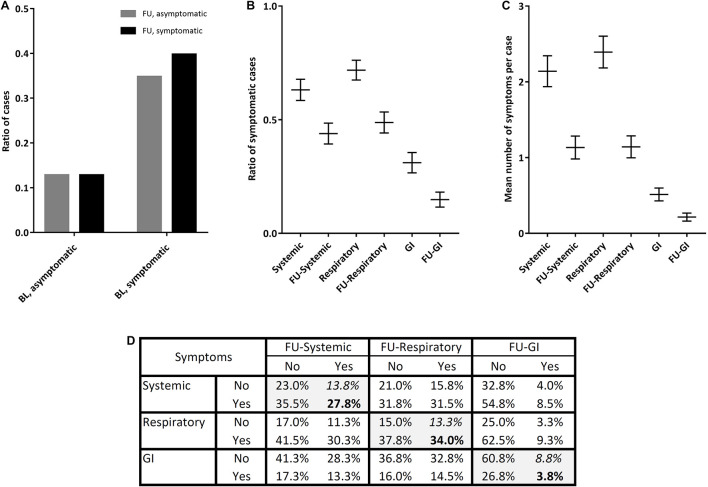
Alterations in the global symptoms as well as the systemic, respiratory, and gastrointestinal (GI) symptoms of 472 COVID-19 patients at the baseline and after 3-month FU. **(A)** The ratio of symptomatic and asymptomatic cases. **(B)** The ratio of symptomatic cases in each symptom class. **(C)** The mean number of symptoms per case in each symptom class. **(D)** The cross-tabulation of symptomatic and asymptomatic subjects at the baseline and FU. (BL: baseline, FU: follow-up, and GI: gastrointestinal).

The frequency of symptomatic subjects decreased from 74.6% at the baseline to 52.5% after 3 months; and, notably, 12.8% of subjects flipped from asymptomatic to symptomatic (Figure 2A). Regarding symptom classes, the frequency of symptomatic subjects decreased from 63.1 to 43.9% in systemic class, from 71.8 to 48.8% in respiratory class, and from 31.1 to 14.9% in GI class (Figure 2B); and the mean count of symptoms per case declined more or less similarly (Figure 2C). The most and least flipping symptom classes were systemic (13.8%) and GI (8.8%) symptoms, respectively (Figure 2D).

Those with blood group A were found to be more frequently flipping to symptomatic in the GI (MD: 28.0%, *P* = 0.007) and respiratory (MD: 23.0%, *P* = 0.010) tracts, as compared with other ABO blood groups. Furthermore, they had more frequent sustaining GI (MD: 24.0%, *P* = 0.005) and respiratory (MD: 11.0%, *P* = 0.036) symptoms. Sustaining systemic symptoms were also seen to be linked to the male sex (MD: 14.0%; *P* = 0.008), and patient’s height (MD: 2.04 cm, *P* = 0.045). On the other hand, those who flipped to asymptomatic at FU did not show any discrepancy in IgM or IgG sustaining as compared with those who remained symptomatic.

Neither at the baseline (*P* = 0.068) nor in the FU (*P* = 0.110), IgM levels were significantly correlated to age (Supplementary Figures 1A,B), while both the baseline (*r*: 0.23, *P* < 0.001) and the FU (*r*: 0.29, *P* < 0.001) IgG levels were higher in older participants (Supplementary Figures 1C,D). Although there was no correlation between sex and the baseline IgM levels, FU IgM levels were significantly higher in men than in women (Supplementary Figure 1E). Furthermore, both the baseline and FU IgG levels were significantly higher in men (Supplementary Figure 1E). The same sex and age associations were found among the sex-stratified subgroups of subjects younger and older than 50 years (Supplementary Figure 1F).

Assessment of the baseline IgM levels did not show any significant differences among ABO blood groups, while the baseline IgG level was significantly lower in the blood group AB (mean [±SD]: 8.46 [±1.01]) as compared with the blood groups A (11.00 [±0.71], *P* = 0.042), B (11.16 [±0.78], *P* = 0.036), and particularly O (11.68 [±0.64], *P* = 0.009) (Supplementary Figure 2). Moreover, the FU IgM level was significantly lower in the blood group AB (0.24 [±0.06]) in comparison with the O (0.55 [±0.11], *P* = 0.009; Supplementary Figure 2). Multivariate analysis identified the overall impact of the baseline characteristics, habits, pre-existing conditions, and the baseline symptoms, on the baseline and FU anti-SARS-CoV-2 IgM and IgG levels. The baseline IgM level was predicted by GI symptoms and pre-existing cirrhosis, while the FU IgM level was just determined by the male sex (Table 2). On the other hand, while both the baseline and FU IgG levels were predicted by age, male sex, and positive chest CT-scan, the baseline IgG level was also correlated with the time (days) passed since the diagnosis (Table 2).

**TABLE 2:** Correlations of the baseline characteristics, habits, pre-existing conditions, and baseline symptoms of COVID-19 patients with the baseline and 3-month follow-up (FU) anti-SARS-CoV-2 IgM and IgG levels.

	**Anti-SARS-CoV-2 IgM antibody level**								**Anti-SARS-CoV-2 IgG antibody level**							
	**Baseline**				**Follow-up**				**Baseline**				**Follow-up**			
	**Univariate**		**Multivariate**		**Univariate**		**Multivariate**		**Univariate**		**Multivariate**		**Univariate**		**Multivariate**	
**Predictor**	***r***	***P***	***r***	***P***	***R***	***P***	***r***	***P***	***R***	***P***	***r***	***P***	***r***	***P***	***r***	***P***
Age	0.085	6.8e−2	–	–	0.075	1.1e−1	–	–	**0.228**	6.8e−7	**0.304**	6.6e−3	**0.289**	2.1e−10	**0.273**	9.8e−3
Male sex	0.006	9.0e−1	–	–	**0.150**	1.1e−3	**0.150**	1.1e−3	**0.185**	5.0e−5	**0.310**	5.8e−3	**0.162**	4.1e−4	**0.422**	1.5e−4
Days since diagnosis	0.038	4.1e−1	–	–	0.043	3.5e−1	–	–	**0.150**	1.1e−3	**0.235**	3.5e−2	**0.133**	3.7e−3	–	–
Blood group AB	−0.056	2.9e−1	–	–	−0.066	2.0e−1	–	–	**−0.123**	1.8e−2	–	–	−0.016	7.6e−1	–	–
Body mass index	0.005	9.2e−1	–	–	−0.061	2.1e−1	–	–	0.089	6.7e−2	–	–	0.088	6.9e−2	–	–
Smoking	0.044	3.7e−1	–	–	0.000	1.0e+0	–	–	−0.064	1.9e−1	–	–	−0.035	4.8e−1	–	–
Alcohol use	−0.048	3.3e−1	–	–	0.002	9.6e−1	–	–	0.018	7.2e−1	–	–	0.071	1.5e−1	–	–
Opium use	−0.008	8.7e−1	–	–	−0.039	4.3e−1	–	–	−0.019	7.0e−1	–	–	−0.053	2.8e−1	–	–
Positive chest CT-scan	0.045	7.4e−1	–	–	0.149	2.7e−1	–	–	**0.363**	5.5e−3	**0.248**	2.5e−2	**0.442**	5.9e−4	**0.338**	1.9e−3
Systemic symptoms	0.087	7.8e−2	–	–	0.053	2.6e−1	–	–	**0.106**	3.1e−2	–	–	**0.112**	1.7e−2	–	–
Respiratory symptoms	0.022	6.6e−1	–	–	−0.004	9.3e−1	–	–	**0.122**	1.3e−2	–	–	0.015	7.5e−1	–	–
Gastrointestinal symptoms	0.128	8.9e−3	**0.118**	1.5e−2	−0.021	6.6e−1	–	–	**0.153**	1.7e−3	–	–	0.014	7.6e−1	–	–
Pregnancy	**0.178**	1.6e−2	–	–	0.129	8.3e−2	–	–	−0.002	9.8e−1	–	–	−0.016	8.3e−1	–	–
Heart disease	0.090	6.4e−2	–	–	0.007	8.9e−1	–	–	0.098	4.5e−2	–	–	**0.103**	3.5e−2	–	–
Hypertension	−0.013	7.8e−1	–	–	−0.040	4.1e−1	–	–	0.031	5.3e−1	–	–	0.063	2.0e−1	–	–
Lung disease	0.026	5.9e−1	–	–	−0.025	6.1e−1	–	–	0.046	3.5e−1	–	–	**0.140**	4.0e−3	–	–
Asthma	0.019	6.9e−1	–	–	−0.035	4.8e−1	–	–	0.028	5.7e−1	–	–	0.018	7.2e−1	–	–
Diabetes mellitus	0.005	9.2e−1	–	–	−0.002	9.7e−1	–	–	**0.100**	4.1e−2	–	–	**0.156**	1.3e−3	–	–
Fatty liver disease	0.080	1.0e−1	–	–	−0.001	9.8e−1	–	–	0.144	3.2e−3	–	–	**0.139**	4.2e−3	–	–
Cirrhosis	**0.182**	1.8e−4	**0.173**	3.8e−4	−0.015	7.7e−1	–	–	0.052	2.9e−1	–	–	0.015	7.7e−1	–	–
Malnutrition	0.082	9.4e−2	–	–	0.013	7.9e−1	–	–	−0.013	8.0e−1	–	–	0.030	5.4e−1	–	–
Inflammatory bowel diseases	**0.116**	1.7e−2	–	–	−0.033	5.1e−1	–	–	−0.015	7.5e−1	–	–	−0.014	7.8e−1	–	–
Hepatitis B	0.007	8.8e−1	–	–	−0.026	6.0e−1	–	–	−0.039	4.3e−1	–	–	−0.029	5.5e−1	–	–
Autoimmune hepatitis	0.067	1.7e−1	–	–	−0.029	5.5e−1	–	–	0.045	3.6e−1	–	–	−0.028	5.7e−1	–	–
Kidney disease	**0.099**	4.3e−2	–	–	−0.017	7.3e−1	–	–	0.089	7.0e−2	–	–	**0.096**	5.0e−2	–	–

*CT: computed tomography, Ig: immunoglobulin, and SARS-CoV-2: novel severe acute respiratory syndrome coronavirus-2.*

*All *r* values standing significant either in univariate or multivariate analysis were shown as bold.*

**FIGURE 3:**
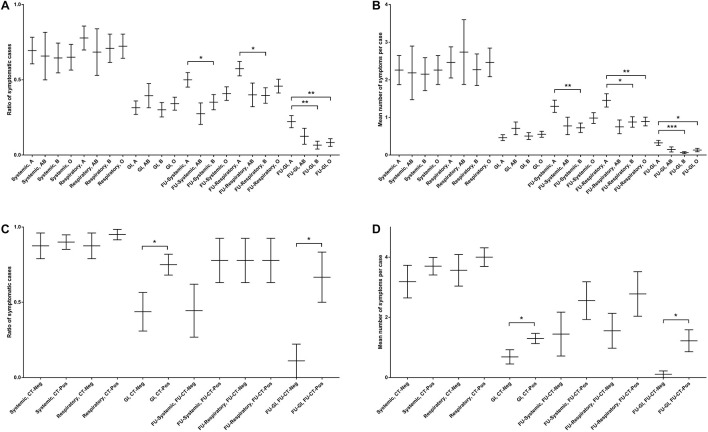
Association of the systemic, respiratory, and GI symptoms at the baseline and after 3-month FU with ABO blood groups and chest computed tomography (CT)-scanning. **(A)** The ratio of symptomatic cases with certain ABO blood groups. **(B)** The mean number of symptomatic cases with particular ABO blood groups. **(C)** The ratio of symptomatic cases with and without a positive lung CT-scanning. **(D)** The mean number of symptomatic cases with and without a positive lung CT-scanning. (CT: computed tomography, FU: follow-up, and GI: gastrointestinal). *unpaired two-tailed Student’s *t*-test, *P* < 0.05. **unpaired two-tailed Student’s *t*-test, *P* < 0.01. ***unpaired two-tailed Student’s *t*-test, *P* < 0.001.

Symptom analysis did not show any significant differences between age- and sex-stratified groups, neither in the distribution of symptomatic cases nor in the count of symptoms per case (Supplementary Figure 3). Furthermore, while no correlations were observed between ABO blood groups and the baseline symptoms, significant associations were found between blood group A and the FU symptoms, particularly GI ones. Subjects with blood group A presented more systemic (MD: 15.0%, *P* = 0.035), respiratory (MD: 18.0%, *P* = 0.012), and GI (MD: 16.0%, *P* = 0.001) symptoms at the FU as compared with those with blood group B (Figure 3A), corresponding to more FU systemic (MD: 0.57, *P* = 0.006), respiratory (MD: 0.57, *P* = 0.011), and GI (MD: 0.26, *P* < 0.001) symptoms per case (Figure 3B). Likewise, the FU GI symptoms were more frequent in blood group A in comparison with blood group O (MD: 14.0%, *P* = 0.004; Figure 3A), corresponding to significantly more GI symptoms per case (MD: 0.19, *P* = 0.017; Figure 3B). Overall, blood group A was found to be correlated with higher risk of respiratory (MD: 43.0%, *P* = 0.010) and GI (MD: 14.0%, *P* = 0.002) symptoms at the FU as compared with the non-A groups (Supplementary Figure 4A), corresponding to more respiratory (MD: 0.59, *P* = 0.003) and GI (MD: 0.21, *P* = 0.003) symptoms per case (Supplementary Figure 4B).

Furthermore, chest CT-scanning results were significantly correlated with GI symptoms at both the baseline and FU assessments. At the baseline, 75.0% of subjects with a positive chest CT-scan had GI symptoms, while just 43.8% of those with a negative CT-scan showed GI symptoms (*P* = 0.042; Figure 3C), corresponding to 1.30 and 0.69 GI symptoms per case in the two CT-scan groups, respectively (*P* = 0.043; Figure 3D). At the FU, 66.7% of participants with a positive chest CT-scan between two assessments reported GI symptoms, while this frequency was 11.1% in those with a negative CT-scan (*P* = 0.015; Figure 3C), corresponding to the mean count of 1.20 and 0.11 GI symptoms per case in the two groups, respectively (*P* = 0.016; Figure 3D).

### Immunoglobulin Sustaining

IgM sustaining was not associated with the systemic, respiratory, or GI symptoms, neither at the baseline nor the FU. Likewise, IgG sustaining was not linked to any baseline or FU symptom classes. However, 97 IgM sustainers had lower BMI (MD: 1.30 kg/m^2^, *P* = 0.002), higher rate of anosmia (MD: 11.5%, *P* = 0.047), and lower rates of cirrhosis (MD: 1.5%, *P* = 0.025) and GI bleeding (MD: 1.6%, *P* = 0.025) in comparison with the IgM decayers. On the other hand, 31 IgG sustainers showed lower rates of the inflammatory bowel diseases (MD: 0.3%, *P* = 0.023), cirrhosis (MD: 1.3%, *P* = 0.025), and GI bleeding (MD: 1.3%, *P* = 0.025) as compared with the IgG decayers. IgG sustainers were also more frequently female (MD: 19.2%, *P* = 0.042) with shorter subsequent diarrhea (MD: 2.8 days, *P* = 0.027).

## Discussion

We performed a longitudinal study on all 472 subjects with a positive anti-SARS-CoV-2 serotyping selected from a national study to assess the natural course of the clinical findings and the serologic response for 3 months, as well as to investigate their relationships and potential affecting factors. Nearly all cases had their clinical symptoms within 3 months of the baseline assessment. While the anti-SARS-CoV-2 IgM seropositivity rate showed an eightfold drop from 192 to 24 seropositive subjects at the FU, the IgG seropositivity rate declined 1.6 folds (from 443 to 276 seropositive subjects); and this is corresponding to mean declines of 6.3 and 2.5 folds in the two antibodies, respectively. The overall frequency of symptomatic subjects declined from 74.5 to 52.5% at the FU, with 12.8% flipping to symptomatic. The respiratory symptoms were the most persistent ones presented at the FU, while the GI symptoms were the least frequent ones. Positive flipping of the GI and respiratory symptoms (To et al., 2020), was linked to the blood group A. Furthermore, sustaining systemic symptoms, potentially indicating the ongoing symptomatic COVID-19 or post-COVID-19 syndrome (Sivan and Taylor, 2020), were linked to male sex and patient’s height, while sustaining GI and respiratory symptoms were linked to the blood group A. The latter observation is consistent with previous studies reporting a higher risk of the disease (odds ratio [OR]: 1.23) (Golinelli et al., 2020) and respiratory failure (OR: 1.45) (Ellinghaus et al., 2020) in patients with blood group A. The ABO blood antigens are found on the normal gastric, antral (Matias-Guiu and Guix, 1988), and intestinal mucosae (Fagerberg et al., 2014), as well as on the normal respiratory epithelia (Bals and Welsch, 1997; Fujitani et al., 2000). Furthermore, angiotensin converting enzyme-2 (ACE2), which is bound by the viral S protein, is known to be highly expressed in the small intestine, duodenum, and gallbladder (Fagerberg et al., 2014); and viral nucleocapsid protein has been visualized in the gastric, duodenal, and rectum glandular epithelial cells (Xiao et al., 2020). Although several studies have already reported the significance of blood group A in more severe COVID-19 disease, to our knowledge, this is the first study proposing a potential implication in long disease, particularly in GI and respiratory systems.

It is proposed that very high levels of the viral ribonucleic acid (RNA) along with occasional detection of cells in stool samples containing subgenomic messenger RNA might indicate active viral replication in the GI tract (Wölfel et al., 2020), very similar to the viable SARS-CoV which can be regularly excreted in stool (Leung et al., 2003). Fecal shedding of the viral RNA has been reported to last between 1 and 33 days after clearance of respiratory samples (Wu et al., 2020). In a study of 95 hospitalized COVID-19 patients, the frequency of positive fecal SARS-CoV-2 was 13.3% higher in those with GI symptoms, though the difference was not statistically significant. Endoscopic examination of six patients with GI symptoms detected SARS-CoV-2 RNA in the esophagus, stomach, duodenum, and rectum specimens of both severe cases, and only in the duodenum of one of the four milder cases (Lin et al., 2020). Besides, in a meta-analysis investigating 6,064 cases, the pooled prevalence of GI symptoms was 15% (95% CI: 10–21%), and patients with GI symptoms had increased risks of acute respiratory distress syndrome (OR: 2.96) and liver injury (OR: 2.71) (Mao et al., 2020). Intriguingly, those subjects with GI symptoms have been reported to have 50% more family clustering as compared with those without the symptoms (Jin et al., 2020).

Although several factors were correlated to the immunoglobulin levels in univariate analyses, a few independently predicted the immunoglobulin levels in multivariate models. Overall, the baseline IgM was associated with GI symptoms and pre-existing cirrhosis, indicating the importance of the GI involvement in acute phase immune response. Furthermore, both the baseline and FU IgG levels were correlated with age, male sex, and positive chest CT-scan. These observation appear to be in line with the facts that COVID-19 is more severe in elderly (Cummings et al., 2020) men (Clark et al., 2020; Huang et al., 2021), and those with a more severe form of the disease develop higher levels of the antibodies (Zhang et al., 2020; Zhao et al., 2020). The finding that the positive chest CT-scan was linked with the baseline and FU GI symptoms rather than with the respiratory symptoms might indicate a close relationship between the lung and GI tract involvement in COVID-19. As previously reported, 84–97% of patients presenting at least one GI symptom have concurrent respiratory symptoms (Luo et al., 2020; Pan et al., 2020).

The presence of anti-spike/nucleocapsid IgG has been correlated with an eightfold lower risk of SARS-CoV-2 reinfection in 6 months (Lumley et al., 2021). Furthermore, Chen and colleagues reported faster symptom resolution in those with sustaining anti-SARS-CoV-2 IgG production (Chen et al., 2020). In contrast, we could not detect any difference in symptom resolution among those with sustaining IgM or IgG after a 3-month FU of a quite larger sample size. However, it cannot be excluded that a more extended and more frequent serologic testing might lead to different results. We observed that IgG sustainers were more commonly women with shorter subsequent diarrhea. Moreover, we found that IgM sustainers were leaner and more anosmic. Given the fact that lower BMI (Gao et al., 2020) and anosmia (Foster et al., 2020; Talavera et al., 2020) are linked to the lower risk of severe COVID-19, this observation might also imply a protective role for sustaining IgM, although it still needs further investigation.

*Limitations*. The diagnostic ability of ELISA kits and limited recruitment, particularly from high-risk occupations, due to the lockdown potentially limited our observation. Furthermore, the anti-SARS-CoV-2 serological tests have shown variable degrees of correlation with the potentially prognostic viral neutralization tests (Szabó et al., 2021) and the lack of the latter in our study makes the interpretation of SARS-CoV-2 antibody levels complicated.

In summary, we performed a longitudinal study on 472 subjects with seropositivity against SARS-CoV-2. Asymptomatic subjects comprised about one-fourth of the population at the baseline, and half of them flipped to symptomatic at the FU. Blood group A was a consistent predictor of both sustaining and flipping of the symptoms in GI and respiratory tracts. Both the baseline and FU IgG levels were correlated with age, male sex, and positive chest CT-scan. We found several factors predicting the sustaining of IgM (anosmia and lower BMI) or IgG (female sex). Our study indicates how clinical and serologic findings in COVID-19 change over time and in relation to each other, and dissects the factors affecting these dynamics.

## Data Availability Statement

The raw data supporting the conclusions of this article will be made available by the authors, without undue reservation.

## Ethics Statement

The studies involving human participants were reviewed and both the study proposal and protocol were approved by the Ethics Committee of the Tehran University of Medical Sciences (reference number: IR.TUMS.VCR.REC.1399.308). The ethical committee performed the approval anonymously. The patients/participants provided their written informed consent to participate in this study.

## Author Contributions

AN, HP, AH, AV-M, MZ, and FR contributed to the conceptualization. MD and MS performed the analysis. AN, HP, AS, and SA drafted, revised, and prepared the manuscript. AS, MS, ZM, FM-G, FJ, GR, AH, RG, KS-h, AA, MM, AB, SG, AD, AV-M, MZ, FR, SA, MD, and RM took part in reviewing the manuscript. ZM, FM-G, FJ, and GR finalized and prepared the manuscript for submission. RM provided the resources and supervised the project. All authors contributed to the article and approved the submitted version.

## Conflict of Interest

The authors declare that the research was conducted in the absence of any commercial or financial relationships that could be construed as a potential conflict of interest.

## Publisher’s Note

All claims expressed in this article are solely those of the authors and do not necessarily represent those of their affiliated organizations, or those of the publisher, the editors and the reviewers. Any product that may be evaluated in this article, or claim that may be made by its manufacturer, is not guaranteed or endorsed by the publisher.

## Footnotes

1
https://coronavirus.jhu.edu/map.html
