# Supplementary material for: Dynamics of the COVID-19 Clinical Findings and the Serologic Response
Source: Front Microbiol. 2021 Oct 7;12:743048. doi: 10.3389/fmicb.2021.743048 (PMC8529145; doi:10.3389/fmicb.2021.743048)
Supplement: Supplementary file 1 [file Table_1.DOCX]

**Dynamics of the COVID-19 Clinical Findings and the Serologic Response-Sup**

Ahmadreza Niavarani^1^†, Hossein Poustchi^2-3^†, Amaneh Shayanrad^2^, Maryam Sharafkhah^2^, Zahra Mohammadi^2^, Fariborz Mansour-Ghanaei^4^, Farahnaz Joukar^4^, Gholamreza Roshandel^5^, Ahmad Hormati^6-7^, Reza Ghadimi^8^, Khosro Sadeghniiat-haghighi^9^, Alireza Abdollahi^10^, Masoud Mardani^11^, Ayad Bahadorimonfared^12^, Shahla Ghanbari^13^, Alireza Delavari^1^, Abbas Vosoogh-Moghaddam^14^, Mohammad Zamani^15^, Farzin Rozafzaei^2^, Saba Alvand^2^*,* Maryam Darvishian^16^, Reza Malekzadeh^1,2,3,17^‡

1. Digestive Oncology Research Center, Digestive Diseases Research Institute, Shariati Hospital, Tehran University of Medical Sciences, Tehran, Iran.
2. Liver and Pancreatobiliary Diseases Research Center, Digestive Diseases Research Institute, Shariati Hospital, Tehran University of Medical Sciences, Tehran, Iran.
3. Sasan Alborz Research Center, Masoud clinic, Tehran, Iran.
4. Division of Gastroenterology & Hepatology Director, Gastrointestinal & Liver Diseases Research Center (GLDRC), Guilan University of Medical Sciences (GUMS), Rasht, Iran.
5. Golestan Research Center of Gastroenterology and Hepatology, Golestan University of Medical Sciences, Gorgan, Iran.
6. Gastrointestinal and Liver Diseases Research Center, Iran University of Medical Sciences, Tehran, Iran.
7. Gastroenterology and Hepatology Disease Research Center, Qom University of Medical Science, Qom, Iran.
8. Social Determinants of Health Research Center, Health Research Institute, Babol University of Medical Sciences, Babol, Iran.
9. Occupational Sleep Research Center, Tehran University of Medical Sciences, Tehran, Iran.
10. Department of pathology, School of Medicine, Imam Hospital complex, Tehran University of Medical Sciences, Tehran, Iran.
11. Infectious Diseases and Tropical Medicine Research Center, Shahid Beheshti University of Medical Sciences, Tehran, Iran.
12. Department of Health & Community Medicine, Faculty of Medicine, Shahid Beheshti University of Medical Sciences, Tehran, Iran.
13. Department of Health Education and Health Promotion, Deputy for Health affairs, Shahid Beheshti University of Medical Sciences, Tehran, Iran.
14. Community Medicine Specialist, Associate Professor, Governance and Health Research Group, Neuroscience Research Institute, Tehran University of Medical Sciences and health services, Tehran, Iran.
15. Digestive Diseases Research Center, Digestive Diseases Research Institute, Tehran University of Medical Sciences, Tehran, Iran.
16. Cancer Control Research, BC Cancer Research Centre, Vancouver, BC, Canada.
17. Digestive Diseases Research Center, Digestive Diseases Research Institute, Shariati Hospital, Tehran University of Medical Sciences, Tehran, Iran

† Co-first authors with equal contribution.

‡ Corresponding author.


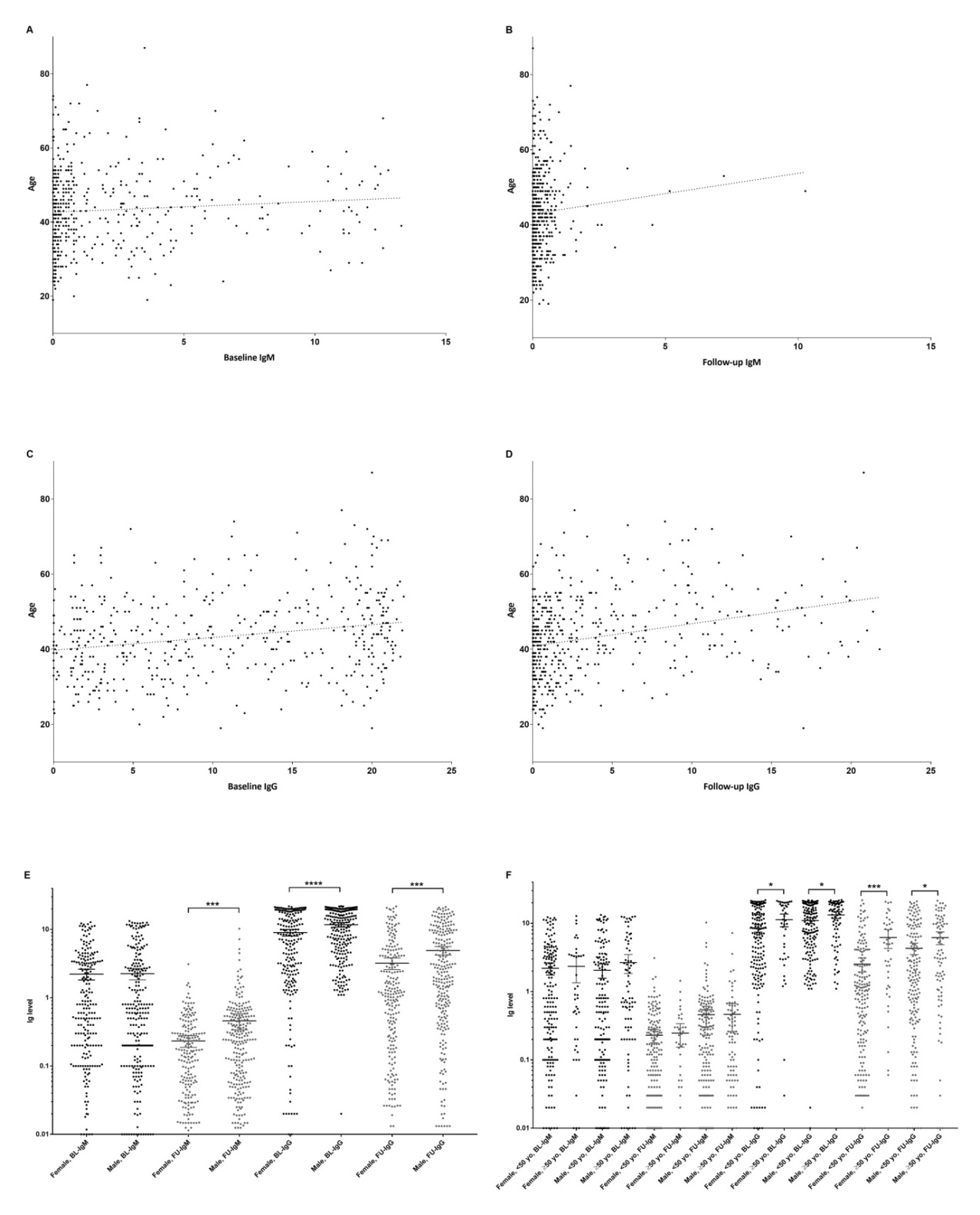


**Figure S1.** Association of the anti-SARS-CoV-2 IgM and IgG levels at the baseline and after 3-month follow-up with age (A-D, F) and sex (E, F).

(BL: baseline, FU: follow-up, Ig: immunoglobulin, yo: years old)

^*^unpaired two-tailed Student’s t-test, *P* < 0.05.

^***^unpaired two-tailed Student’s t-test, *P* < 0.001.

^****^unpaired two-tailed Student’s t-test, *P* < 0.0001.


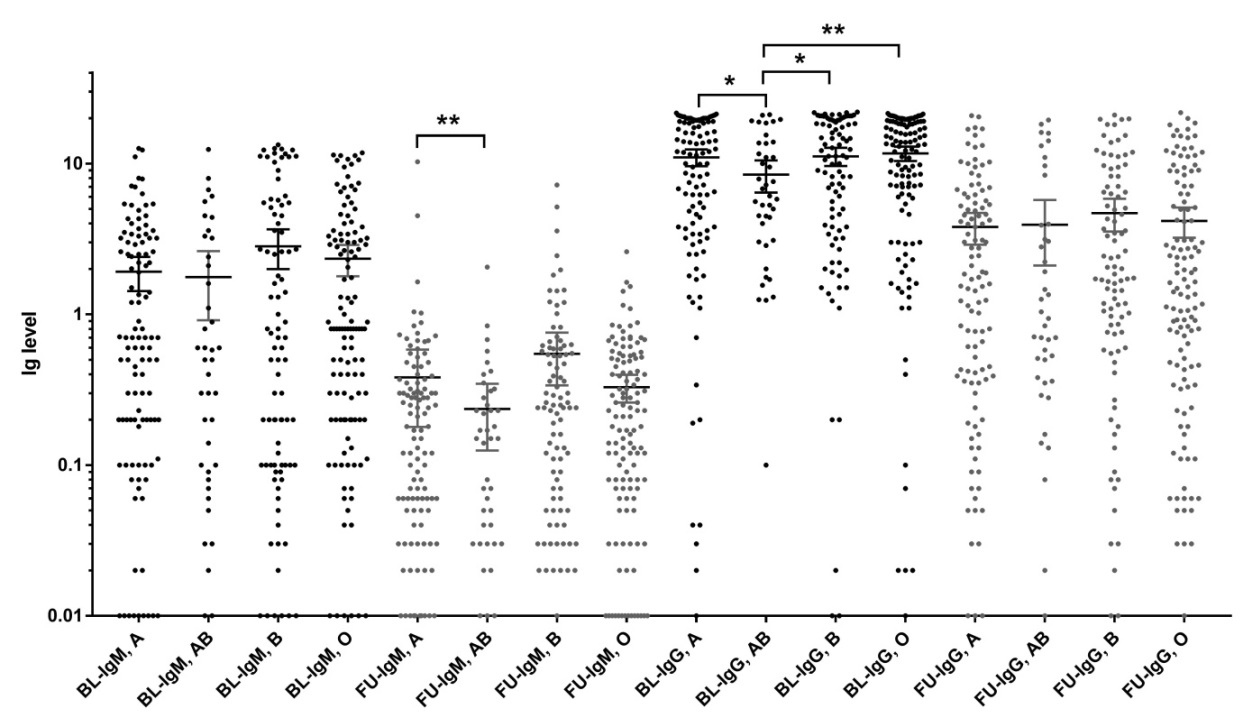


**Figure S2.** Association of the anti-SARS-CoV-2 IgM and IgG levels at the baseline and after 3-month follow-up with ABO blood groups.

(BL: baseline, FU: follow-up, Ig: immunoglobulin)
^*^unpaired two-tailed Student’s t-test, *P* < 0.05.
^**^unpaired two-tailed Student’s t-test, *P* < 0.01.


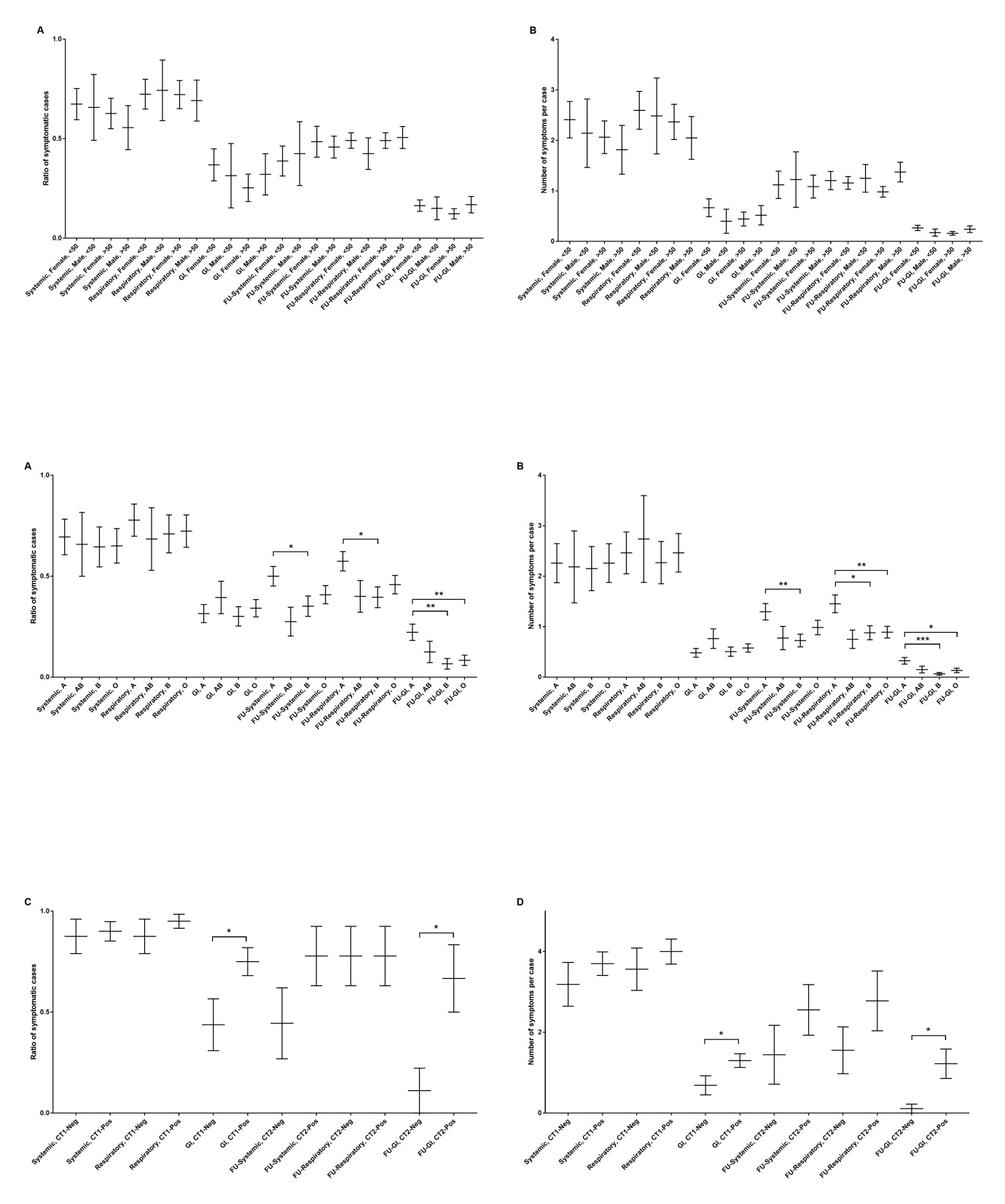
**Figure S3.** Association of the systemic, respiratory, and gastrointestinal symptoms at the baseline and after 3-month follow-up with age and sex. **A.** The ratio of symptomatic cases. **B.** The mean number of symptoms per case.

(FU: follow-up, GI: gastrointestinal)


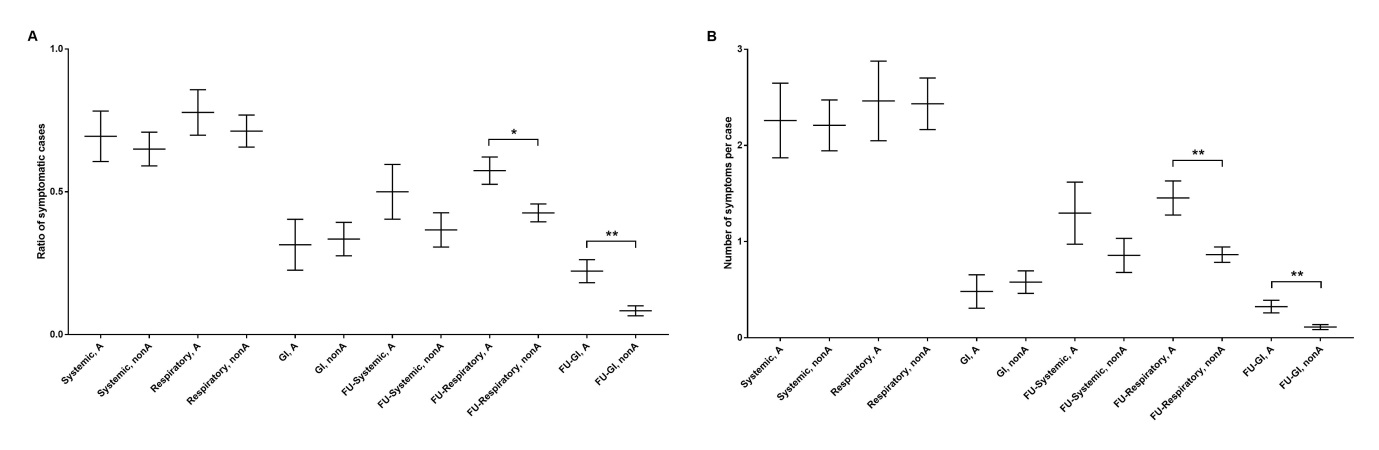


**Figure S4.** Association of the systemic, respiratory, and gastrointestinal symptoms at the baseline and after 3-month follow-up with blood group A compared to non-A blood groups. **A.** The ratio of symptomatic cases. **B.** The mean number of symptoms per case.

(FU: follow-up, GI: gastrointestinal)

^*^unpaired two-tailed Student’s t-test, *P* < 0.05.

^**^unpaired two-tailed Student’s t-test, *P* < 0.01.
